# New Ataxic *Tottering-6j* Mouse Allele Containing a *Cacna1a* Gene Mutation

**DOI:** 10.1371/journal.pone.0044230

**Published:** 2012-08-31

**Authors:** Weidong Li, Ying Zhou, Xiaoli Tian, Tae Yeon Kim, Namiko Ito, Kaori Watanabe, Akiko Tsuji, Kimie Niimi, Yo Aoyama, Takashi Arai, Eiki Takahashi

**Affiliations:** 1 Bio-X Institutes, Key Laboratory for the Genetics of Developmental and Neuropsychiatric Disorders (Ministry of Education), Shanghai Jiao Tong University, Shanghai, People’s Republic of China; 2 Division of Pulmonary and Critical Care Medicine, David Geffen School of Medicine, University of California Los Angeles, Los Angeles, California, United States of America; 3 Research Resources Center, RIKEN Brain Science Institute, Saitama, Japan; St. Jude Children's Research Hospital, United States of America

## Abstract

Voltage-gated Ca^2+^ (Ca_v_) channels control neuronal functions including neurotransmitter release and gene expression. The *Cacna1a* gene encodes the α1 subunit of the pore-forming Ca_v_2.1 channel. Mice with mutations in this gene form useful tools for defining channel functions. The recessive ataxic *tottering-6j* strain that was generated in the Neuroscience Mutagenesis Facility at The Jackson Laboratory has a mutation in the *Cacna1a* gene. However, the effect of this mutation has not been investigated in detail. In this study, mutation analysis shows a base substitution (C-to-A) in the consensus splice acceptor sequence linked to exon 5, which results in the skipping of exon 5 and the splicing of exon 4 directly to exon 6. The effect of this mutation is expected to be severe as the expressed α1 subunit protein lacks a significant part of the S4–S5 linker, S5, and part of S5–S6 linker in domain I. *Tottering-6j* mice display motor dysfunctions in the footprint, rotating rod, and hind-limb extension tests. Although cytoarchitecture of the mutant brains appears normal, tyrosine hydroxylase was persistently expressed in cerebellar Purkinje cells in the adult mutant mice. These results indicate that *tottering-6j* is a useful model for functional studies of the Ca_v_2.1 channel.

## Introduction

Voltage-gated Ca^2+^ (Ca_v_) channels play an important role in the regulation of diverse neuronal functions which are attributed to elevated intracellular Ca^2+^ concentrations [1], [2]. The pore-forming α1 subunit functions as a voltage sensor and is capable of generating channel activity [3]. The α1 subunit consists of four homologous transmembrane domains (I-IV), each containing six transmembrane spanning α-helices (S1–S6) [4], [5]. The four domains are connected through cytoplasmic linkers, and both the C- and N-termini are cytoplasmic and interact with regulatory proteins [6], [7].

Mutations within the α_1_ subunit (Ca_v_2.1α_1_) gene from the Ca_v_2.1 channel have been identified [8], [9]. In humans, these mutations cause several autosomal dominant neurological defects, including familial hemiplegic migraine (FHM), episodic ataxia type-2 (EA2), and spinocerebellar ataxia (SCA6) [10]. To examine the function and disease processes of the Ca_v_2.1 channel, mouse genetic approaches can be useful. Mice with mutations in the Ca_v_2.1α_1_ *Cacna1a* gene have been reported, and include the FHM1 model strains (R192Q and S218L knockin mice) [11], [12], a SCA6 model strain carrying additional CAG repeats in the *Cacna1a* locus of the knockin mice [13], and a knockout strain lacking Ca_v_2.1 currents [14]. It has also been reported that in spontaneous or chemically-induced *Cacna1a* mutant strains, dominant mutations were detected in the *tottering-5j* and *wobbly* mice and recessive mutations were detected in the *rocker*, *tottering*, *rolling Nagoya*, *tottering-4j*, and *leaner* mice [15], [16], [17]. In contrast to the heterozygous *tottering-5j* and *wobbly* mice, which showed mild ataxia and had normal life spans, the homozygous *tottering-5j* and *wobbly* mice showed severe ataxia and died prematurely. All of the homozygous recessive mouse mutants developed ataxia and have normal life spans. The chemically induced ataxic *groggy* rat is a recessive *Cacna1a* mutant with a normal life span [18]. *Cacna1a* mutant strain serves as a motor neuron disease model. Cav2.1 channels express at the neuromuscular junction (NMJ) and regulate acetylcholine (ACh) release from motor neurons [19]; abnormal ACh release is the cause of NMJ dysfunction in *tottering* and *rolling Nagoya* [20], [21]. Mice with motor neuron disease displayed clasping behavior in the hind-limb extension test [22], similar to that shown by *rolling Nagoya* mice [23].

We describe here a novel *Cacna1a* gene mutant, the *tottering-6j* mouse, generated in the Neuroscience Mutagenesis Facility at The Jackson Laboratory (MN, USA). The *tottering-6j* mice are a chemically-induced mutant strain produced using ethylnitrosourea (ENU) and show a similar phenotype to the *tottering* mice in the Jackson Laboratory Database (http://jaxmice.jax.org/strain/008623.html). The database showed that the complementation test performed between *tottering* and *tottering-6j* mice indicated that the *tottering-6j* mice have a recessive mutation in the *Cacna1a* gene. However, the exact position of the mutation and the advanced motor behavior of this strain have not been examined. Motor behavior was studied using the footprint [24], traction [25], rotating rod [26], and hind-limb extension [23] tests, all of which are well characterized and reliable. Tyrosine hydroxylase is a key enzyme in the noradrenergic biosynthesis pathway. Its expression is normally transient in a subset of cerebellar Purkinje cells and is not present 40 days postnatally [27]. By contrast, this transient expression persists into adulthood in *tottering* mice [27], [28]. This expression pattern indicates that Ca^2+^ misregulation leading to the responsiveness of the tyrosine hydroxylase promoter and reflecting abnormal Ca^2+^ signaling causes motor dysfunction.

In this study, to characterize aberrant neuronal network in motor function of *tottering-6j* mice, we identified the causative mutation in the *Cacna1a* gene, and examined the poor motor coordination, and the altered tyrosine hydroxylase expression in the cerebellar Purkinje cells of the *tottering-6j* mice.

## Results

### Transcript and Genomic Structure of the *Cacna1a* Gene in *Tottering-6j* Mutant Mice

**Figure 1:**
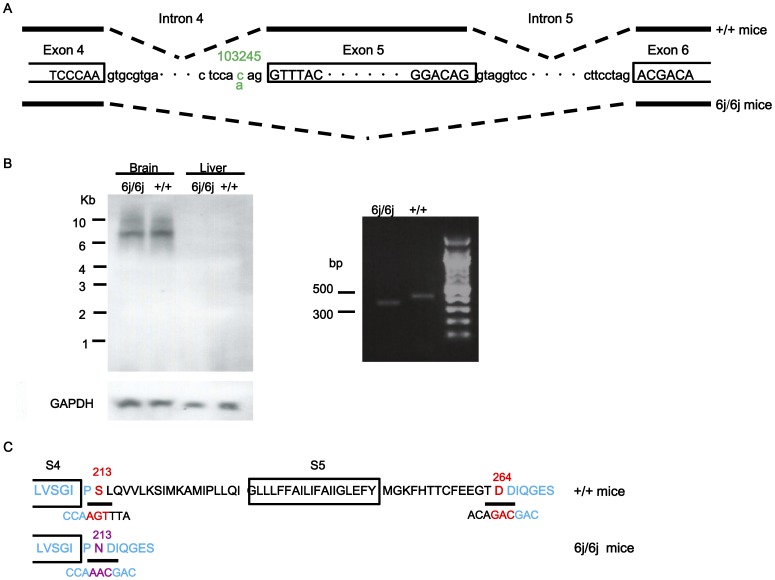
Structure and expression of the *Cacna1a* gene in *tottering-6j* mice. Intron/exon and transcript structure in *6j*/*6j* and +/+ strains is shown (A). Exon sequences are indicated by capital letters in boxes. Intron sequences indicate lowercase. The C-to-A substitution at the nucleotide residue 103245 is indicated in green. Transcripts are indicated below and above as genotypes; these are *6j*/*6j* and +/+ strain, respectively. Splicing events are indicated by dashed lines. Analysis of *Cacna1a* gene expression in *6j*/*6j* (*n = *8) and +/+ (*n = *8) mice by Western blot and RT-PCR (B). Representative protein expression pattern of the *Cacna1a* gene is shown. GAPDH was used to control for total RNA loading. No differences were observed between the *6j*/*6j* and the +/+ mice. Representative RT-PCR results are shown. The fragment from the brain of the *6j*/*6j* mice was smaller compared with that of the +/+ mice. A part of protein sequences of domain I in the Ca_v_2.1α_1_ are shown (C). The protein sequence for the transmembrane segments of S4 and S5 is indicated by the boxes. The upper protein sequence is +/+ and the lower is *6j*/*6j*, respectively. The nucleotide sequence encoding the amino acid is indicated under the protein sequence. The blue highlighting indicates the alternate exons. Red highlighting indicates amino acids encoded across a splice junction. The deleted amino acid region is 213–264. The altered amino acid is 213 is indicated in purple.

Sequencing of the *Cacna1a* genomic DNA from homozygous *tottering-6j* (*6j/6j*) mutants revealed a C-to-A transversion at nucleotide 103245 (Fig. 1A). The mutation is located in the consensus splice acceptor sequence linked to exon 5 and results in the skipping of exon 5, removing 153 bp and splicing directly to exon 6. These mutations were not found in the *Cacna1a* genomic and cDNA from wild-type (+/+) mice. Northern blot analyses did not detect any differences in the brain *Cacna1a* mRNA expression between *6j/6j* and +/+ littermates (Fig. 1B). The RT-PCR fragment generated from the brain showed that a smaller size fragment was detected in *6j/6j* mice (Fig. 1B). The expressed α1 subunit protein in the mutants is predicted to lack part of the S4–S5 linker, S5, and a part of S5–S6 in domain I consisting of amino acids at 213 (serine) to 264 (asparatic acid) and contain a new amino acid at 213 (asparagine) (Fig. 1C). Segregation analysis of the point mutation revealed coinheritance of the mutation and ataxic phenotype in all 42 *6j*/*6j* mice tested, while all 84 heterozygous *tottering-6j* (*6j*/+) and 42+/+ littermates were heterozygote and wild-type, respectively, at the *tottering-6j* locus (data not shown).

### Normal Muscle Strength in *Tottering-6j* Mutant Mice

**Figure 2:**
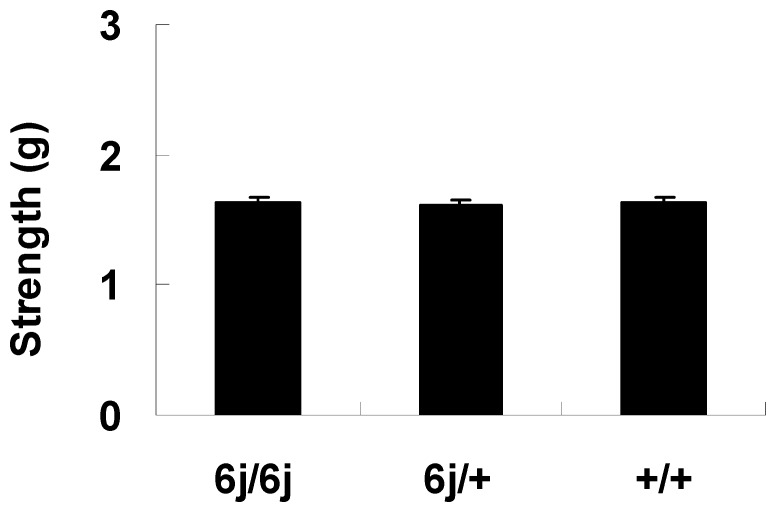
Muscle strength in the grip strength test. The *6j*/*6j* (*n = *10), *6j*/+ (*n = *10), and +/+ mice (*n = *10) were examined in motor behavior tests.

The results from the grip strength test showed the groups did not differ significantly in muscle strength (F(2, 27) = 0.045; *P* = 0.957) (Fig. 2).

### Motor Dysfunctions in *Tottering-6j* Mutant Mice

**Figure 3:**
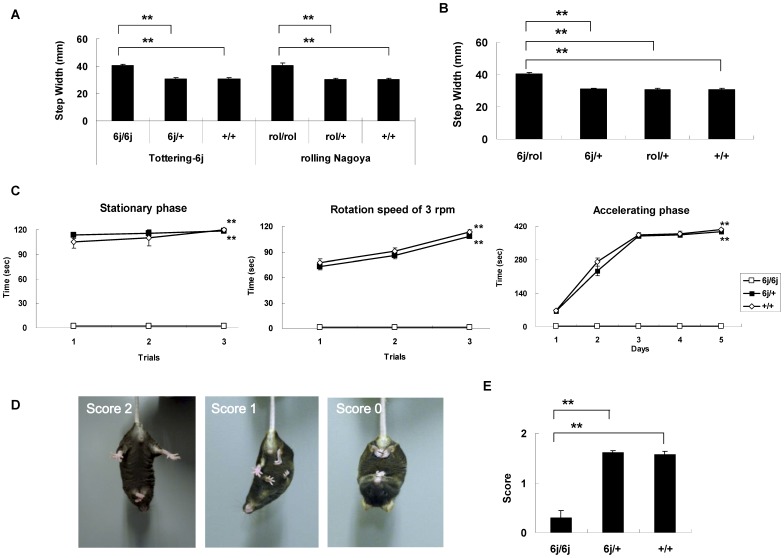
Motor coordination was assessed using the footprint, rotating rod, and hind-limb extension tests. The footprint test was used to examine the walking pattern (A, B). The *tottering-6j* strain including *6j*/*6j* (*n = *15), *6j*/+ (*n = *15), and +/+ mice (*n = *15), and the *rolling Nagoya* strain including *rol*/*rol* (*n = *15), *rol*/+ (*n = *15), and +/+ mice (*n = *15) were used (A). The heterozygous mice including *6j*/*rol* (*n = *15), *6j*/*+* (*n = *15), *rol*/+ (*n = *15), and +/+ (*n = *15) mice were used (B). In the rotating rod test, retention time on the rotating rod was examined in *6j*/*6j* (*n = *10), *6j*/+ (*n = *10), and +/+ mice (*n = *10) (C). In the hind-limb extension test, hind-limb posture was scored as 2, 1, or 0 (D), and the score for each strain is presented (E) for *6j*/*6j* (*n = *10), *6j*/+ (*n = *10), and +/+ mice (*n = *10). **P<0.001 compared to the appropriate control (Tukey's test).

We examined the walking pattern using the footprint test to study ataxic phenotype and to perform the complementation test. There were no significant differences among *6j*/*6j*, *6j*/+ and +/+ mice comparing stride length (left; F(2, 42) = 0.014, *P* = 0.986, right; F(2, 42) = 0.069; *P* = 0.933) (data not shown). However, there was a significant difference when comparing step width (F(2, 42) = 26.802, *P*<0.001) (Fig. 3A). The *6j*/*6j* mice had a larger step width than the *6j*/+ mice (*P*<0.001) and the +/+ mice (*P*<0.001). There were no significant differences between the homozygous *rolling Nagoya*, (*rol*/*rol*), heterozygous *rolling Nagoya*, (*rol*/*+*) and the +/+ mice comparing the stride length (left; F(2, 42) = 0.008, *P* = 0.992, right; F(2, 42) = 0.013; *P* = 0.987) (data not shown). However, there were significant differences in step width (F(2, 42) = 21.845, *P*<0.001) (Fig. 3A). There was a significant difference between the *rol*/*rol* and *rol*/+ mice (*P*<0.001) and between the *rol*/*rol* and the +/+ mice (*P*<0.001). To test for complementarity to a known *Cacna1a* allele, such as the *rolling Nagoya*, *6j*/*+* mice were crossed with the *rol*/+ mice. The groups did not differ significantly between compound heterozygous (*tottering-6j* × *rolling Nagoya*, *6j*/*rol*), *6j*/*+*, *rol*/+, and +/+ mice when comparing stride length (left, F(3, 56) = 0.004; *P* = 0.999, right, F(3, 56) = 0.004; *P* = 0.991) (data not shown). There were significant differences between the strains when comparing step width (F(3, 56) = 31.180; *P*<0.001) (Fig. 3B). There was a significant difference between the *6j*/*rol* and the *6j*/*+* mice (*P*<0.001), between the *6j*/*rol* and the *rol*/+ mice (*P*<0.001), and between the *6j*/*rol* and the +/+ mice (*P*<0.001).

The *6j*/*6j* mice were not able to maintain balance on a stationary rod (0 rpm) (F(2, 81) = 580.892; *P*<0.001) or during rotation speed of 3 rpm (F(2, 81) = 592.887; *P*<0.001) or in the accelerating phase (F(2, 435) = 871.034; *P*<0.001) (Fig. 3C). There were significant differences between the *6j*/*6j* and the *6j*/+ mice (0 rpm; *P*<0.001, 3 rpm; *P*<0.001, accelerating phase; *P*<0.001) and between the *6j*/*6j and* the +/+ mice (0 rpm; *P*<0.001, 3 rpm; *P*<0.001, accelerating phase; *P*<0.001) (Fig. 3C).

Normally, an extension reflex in the hind-limb is observed when a mouse is suspended in the air by its tail. However, in mice with motor neuron disease, hind-limb retraction is observed more commonly (Fig. 3D). The groups differed significantly in the score (F(2, 29) = 53.926; *P*<0.001) and a significant difference was observed between the *6j*/*6j* and the *6j*/+ mice (*P*<0.001) and between the *6j*/*6j* and the +/+ mice (*P*<0.001) (Fig. 3E).

### Brain Cytoarchitecture and Cerebellar Expression of Tyrosine Hydroxylase in *Tottering-6j* Mutant Mice

**Figure 4:**
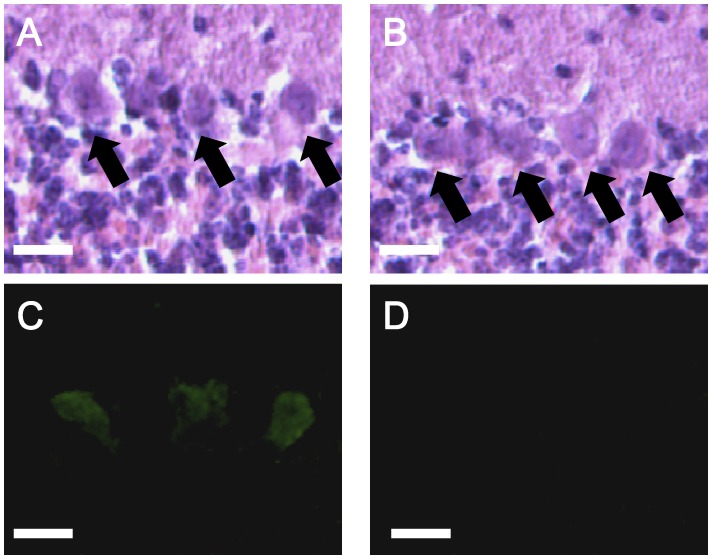
Representative histochemistry in the cerebellum of *tottering-6j* mice. Hematoxylin eosin (HE) staining of *6j/6j* (A) and +/+ (B) cerebella, and tyrosine hydroxylase (TH) staining of *6j/6j* (C) and +/+ (D) cerebella are shown. TH was detected in the Purkinje cells of the *6j/6j* mice (*n = *6) but not in those of +/+ mice (*n = *6). Arrows point to Purkinje cell somata. Scale bar, 20 µm.

Our initial pathology studies involved gross histological examination with serial sections through the whole brain. The cytoarchitecture of the mutant brains appeared normal (data not shown). We investigated the cerebellum more closely to explain the ataxia observed in the mutant. We found a normal cerebellar morphology and cytoarchitecture in the *6j*/*6j* mice compared with the +/+ mice. Tyrosine hydroxylase expression is detected in a subset of the cerebellar Purkinje cells of the *6j*/*6j* mice but not in the +/+ mice at eight weeks of age (Fig. 4).

## Discussion

In this study, we identified the mouse mutant *tottering-6j* as a new allele of the Ca_v_2.1 α_1_ subunit, *Cacna1a* gene using sequence analyses, behavior tests, and histological studies.

**Figure 5:**
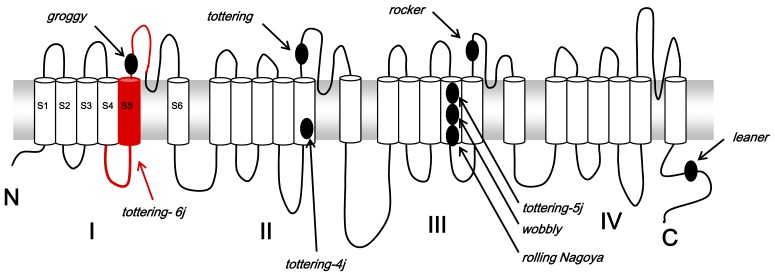
Proposed transmembrane topography of the Cav2.1α1 subunit and positions of known mutations identified in the *Cacna1a* mutant mice and rat. The deletion region including part of the S4–S5 linker, S5, and a part of S5–S6 in domain I of Cav2.1α1 in the *tottering-6j* mice is shown by the red line.

The Jackson Laboratory performed the complementation test between the *tottering* and the *tottering-6j* mice. The result indicated that the *tottering-6j* mice have a recessive mutation in the *Cacna1a* gene. To confirm this result, we used another *Cacna1a* mutant, the *rolling Nagoya* mice [23], [29]. Our complementation and segregation tests also showed that the *tottering-6j* mice have a recessive mutation in the *Cacna1a* allele. Most genes are divided into exons (coding region) and introns (intervening non-coding region). Transcription of genomic DNA creates large pre-RNAs from which the intervening non-coding regions must be precisely removed to create functional mRNAs. Splicing is performed by small nuclear RNAs (snRNAs) and proteins that recognize consensus sequences on the genomic DNA at or near the splice junctions [30]. We identified a CAG to AAG transverse at the 3′ splice acceptor sequence in intron 4, resulting in the skipping of exon 5 and the splicing of exon 4 directly with exon 6. Although the mechanism has remained unclear, the altered sequence is not recognized as a splice acceptor sequence. The expressed α1 subunit protein in the *tottering-6j* mice is predicted to lack part of the S4–S5 linker, S5, and a part of S5–S6 in domain I (Fig. 5). Because the S5 and S6 segments and the membrane-associated P-loop connecting them in the α1 subunit form the pore lining of the ion channel [31], at least, part of the pore lining of the Ca_v_2.1 channel would be dysfunctional.

Ca_v_2.1α_1_ is a molecular complex comprising several proteins [6], [7]. The intracellular N- and C-termini and the cytoplasmic loops connecting domains I–IV are important for interaction with other proteins including the β subunit of the channel that binds to the I–II loop, synaptic proteins that interact at the synaptic protein interaction (synprint) site found in the II-III loop, and G protein βγ heterodimers (Gβγ) that interact at three sites on the N-terminus, I–II loop and C-terminus. Because there was not a frame shift after the deletion in the *tottering-6j* mice, the binding site for these proteins would be intact. It has been reported that most Ca_v_2.1 knockout mice do not survive past weaning [14]. However, most of the *tottering-6j* mice had normal life spans (data not shown). If there was a frame shift after the deletion site, the *tottering-6j* mice would show similar phenotypes to the knockout mice.

Given the pivotal role of the Ca_v_2.1 channel in controlling neurotransmitter production and release, defects in the structure of the presynaptic Ca_v_2.1 channel result in aberrant synaptic signaling leading to various patterns of neural network dysfunction and behavior disorders. In the behavior tests, *tottering-6j* mice clearly suffer from ataxia without the loss of muscle strength. All of the homozygous recessive *Cacna1a* mice developed ataxia, ranging from mild in the *rocker*, *tottering*, and *rolling Nagoya* mice to severe ataxia in the *leaner* mice [9]. The mutation identified in the *tottering-6j* mice is similar to the *rocker*, *tottering*, or *groggy* mutation in the structural location of the Cav2.1α1 subunit (Fig. 5). Particularly, the *groggy* rats have an amino acid substitution located in the S5–S6 linker in domain I [18]. The *groggy* rats show mild ataxia resembling the *rocker* or *tottering* mice rather than the *rolling Nagoya* mice. In our sturdy, the *tottering-6j* mice exhibited similar walking patterns to the *rolling Nagoya* mice. The *rolling Nagoya* mice have a mutation in the voltage-sensing S4 segment in the domain III [32], and *leaner* mice have a mutation in a splice donor consensus sequence, which results in an altered C-terminal sequence [27] (Fig. 5). The reduction in amplitude of the P-type Ca^2+^ current was greater in the Purkinje cells of the *leaner* mice (60%) [33] compared with the *tottering* and *rolling Nagoya* mice (40%) [32]. These results indicate that the threshold dose for ataxia differs for different mutants. The Purkinje cells of 8-week-old *tottering-6j* mice showed tyrosine hydroxylase expression. Although tyrosine hydroxylase expression was no longer detected in adult *rocker* mice [34], ectopic tyrosine hydroxylase expression occurred in the other recessive *Cacna1a* mouse mutants including *tottering* [27], *rolling Nagoya* [35], *tottering-4j* [17], and *leaner* [27] mice. Tyrosine hydroxylase is normally expressed only during development; thus, ectopic tyrosine hydroxylase expression in *Cacna1a* mutants may indicate delayed neuronal maturation. Because the Ca^2+^ concentration in Purkinje cells is an important determinant of tyrosine hydroxylase expression [36], [37], ectopic tyrosine hydroxylase expression is likely the direct result of Ca^2+^ dysregulation due to Cav2.1 dysfunction. The *rocker* mice exhibited the mildest ataxia of the homozygous recessive *Cacna1a* mutants [34]. It may be that the lower magnitude change in Ca^2+^ exhibited in the *rocker* mice results in the mild ataxia and inability to downregulate tyrosine hydroxylase. Electrophysiological examination of the Ca^2+^ flux in Purkinje cells may be helpful in evaluating the validity of this hypothesis. The cerebellum of *Cacna1a* mutants has been reported to show significant synaptic changes. An altered synaptic pattern between cerebellar parallel fibers and Purkinje cells has been reported in adult *tottering* and *leaner* mice [37]. *Rolling Nagoya* mice show abnormally shaped Purkinje cell dendritic spines and single parallel fiber varicosities making multiple synaptic contacts, which were not observed in the wild type [38], suggesting that aberrant branching may be present in the Purkinje cells of *tottering-6j* mice. The present study found that although the *tottering-6j* mutant mice showed normal muscle strength in the grip-strength test, they exhibited an abnormal hind-limb extension reflex. Mice with motor dysfunction displayed reduced hind-limb extension and abnormal ACh receptor expression at the NMJ [22]. These results indicate that the *tottering-6j* mice have a deficit in ACh release at the NMJ. We plan to conduct electorophysiological, ultrastructural, and morphological studies; however, a detailed comparison of allelic variants would be helpful in clarifying the relationships among the many different structural, physiological, and synaptic abnormalities, and the observed behavioral deficits and the understanding of the channel functions.

In summary, the recessive ataxic *tottering-6j* strain contains a mutation in the *Cacna1a* gene. The mutation analysis shows a base substitution (C-to-A) in the consensus splice acceptor sequence linked to exon 5, resulting in the skipping of exon 5 and the deletion of a part of the pore lining of the α_1_ subunit of Ca_v_2.1 channel. *Tottering-6j* mice display motor dysfunctions in the footprint, rotating rod, and hind-limb extension tests. Although gross cytoarchitecture of the mutant brains appears normal, tyrosine hydroxylase was expressed in cerebellar Purkinje cells in the mice at eight weeks of age. These results indicate that the *tottering-6j* strain is useful model for functional studies of the Ca_v_2.1 channel.

## Materials and Methods

### Ethics Statement

The research was conducted in accordance with the Declaration of the Helsinki and was approved by the Animal Experiments Committee of RIKEN Brain Science Institute. All animals were cared for and treated humanely in accordance with the Institutional Guideline for Experiments using Animals (Approved ID: No. H24-2-206).

### Animals

The *tottering-6j* mouse strain with the C57BL/6J and BALB/cByJ mixed genetic background was provided by the Jackson Laboratory. *Tottering-6j* mice backcrossed to C57BL/6J mice for 10 generations produced *tottering-6j* mice with a C57BL/6J genetic background. We used the *rolling Nagoya* mouse strain [23] with a C57BL/6J genetic background (backcross generations; *N* = 10) for complementation tests. The mice were allowed *ad libitum* access to water and food pellets (CRF-1; Oriental Yeast, Tokyo, Japan) and kept at room temperature (23±1°C) and 55±5% humidity under a 12∶12-h light-dark cycle (light from 8∶00 am to 8∶00 pm).

### Mutation Analysis of the Transcript and Genomic Structure of the *Cacna1a* Gene

The mice were anesthetized with isofluorane and killed by decapitation, and whole brains were dissected. Total RNA was isolated from brains of 24 eight-week-old homozygous *tottering-6j* (*6j*/*6j*) and 24 wild-type (+/+) mice using TRIzol reagent, according to the manufacture’s protocol (Invitrogen, ON, Canada). The first-strand complementary DNA (cDNA) was synthesized by oligo(dT) priming (SuperScript First-Strand Synthesis System; Invitrogen, ON, Canada). Reverse transcriptase-polymerase chain reaction (RT-PCR) primers were designed to create fourteen 400- to 800-bp fragments covering the entire 7929-bp messenger RNA (mRNA) sequence of *Cacna1a*. The RT-PCR products were sequenced using an automated sequencer (ABI Prism 3730; Applied Biosystems, CA, USA). Alternation in the transcript structure was discovered in Ca_v_2.1α_1_ using the C57BL/6J, and BALB/cByJ, and database sequence of *Cacna1a* cDNA (GenBank ID: NM_007578). The following PCR primers (Forward: 5′-CCTCTCTGTGGGTACACATAT-3′, reverse: 5′-GGGAATACTGAATTCAGGATT-3′) were used to confirm the mutation position in the fragment consisting of the nucleotides 99881 (in intron 3) - 108044 (in intron 6) of the mouse genomic *Cacna1a* DNA (GenBank ID: NC_000074) from spleens of 24 eight-week-old *6j*/*6j* and 24+/+ mice. Northern blotting was used to examine the transcript levels, where 10 µg of the total RNA isolated from the brain and the liver of eight-week-old *6j*/*6j* and +/+ mice was used for blot hybridization with a DIG labeled probe consisting of nucleotides 3864–4664 of the mouse *Cacna1a* cDNA (GenBank ID: NM_007578). For RT-PCR analysis, the following PCR primers (Forward: 5′-TCCTACCTGAGGAATGGCTGGAAC-3′, reverse 5′-CAGCCTTCCATGGTGATGCACTCC-3′) were used to identify the genotypes with the wild-type 473-bp or mutant-type 320-bp fragments consisting of nucleotides 493 (in exon 4) - 965 (in exon 6) of the mouse *Cacna1a* cDNA (GenBank ID: NM_007578) from brains.

### Motor Behavior Tests

The mice, including eight-week-old male *6j*/*6j*, heterozygous *tottering-6j* (*6j*/*+*), homozygous *rolling Nagoya*, (*rol*/*rol*), heterozygous *rolling Nagoya*, (*rol*/*+*), compound heterozygous (*tottering-6j* × *rolling Nagoya*, *6j*/*rol*), and +/+ mice were subjected to motor behavior studies. In the footprint test, black ink was applied to the hind paws of each mouse and they were then placed in a narrow alley (9 × 25 × 10 cm) on white paper. Stride length and step width were measured. The black ink used for the footprint analysis was non-toxic. The footprint test was conducted between 10∶00 am and 12∶00 pm. In the traction test, the grip strength of each mouse was measured using a traction apparatus (Ohara & Co., Ltd., Tokyo, Japan). Each mouse was made to grasp the attached bar (1 mm diameter) with the forepaws and was slowly pulled back by its tail. The maximum tension (in g) before release was recorded and normalized to body weight. The traction test was conducted between 11∶00 am and 11∶30 pm. In the rotating rod test, motor coordination was assessed with a rotating rod apparatus (Ugo Basile RotaRod Treadmills, Model 7650; Ugo Basile S.R.L., Comerio, Italy). The mice were first placed on the stationary rod (0 rpm) for three trials, followed by three trials at a rotation speed of 3 rpm. Latency until a fall occurred was monitored for 120 s and the intra-trial intervals for each animal were greater than 20 min. The rotation of the rotarod was accelerated from 3 to 30 rpm over 300 s at a constant rate, and the rotation speed of 30 rpm was maintained for 120 s. Mice were trained for five days and received three trials per day, with an interval of 1 h between trials. The time taken for each mouse to maintain balance on the rotarod was measured. The rotating rod test was conducted between 1∶00 am and 4∶00 pm. In the hind-limb extension test, the mice were suspended by the tail and the extent of hind-limb extension was observed during 10 s. A score of 2 corresponded to a normal extension reflex in both hind-limbs, with splaying of toes. A score of 1 corresponded to an extension reflex in only one hind-limb or extension of both hind-limbs, without splayed toes. A score of 0 corresponded to clasping behavior with both hind-limbs. The hind-limb extension test was conducted between 1∶00 am and 2∶00 pm. All behavioral analyses were conducted by a well-trained experimenter who was blinded to the mouse genotypes. The mice were moved into the behavioral testing room at least 1 h before testing. The data are presented as the mean ± standard error of the mean (SEM). Statistical analyses were conducted using Excel Statistics 2006 (SSRI, Tokyo, Japan). The data were analyzed using an analysis of variance (ANOVA). Tukey's *post hoc* test between groups was performed when appropriate. The results were considered significant at a 5% or lower probability of error.

### Histochemistry

At 8 weeks of age, animals were anesthetized using sodium pentobarbitone and perfused transcardially with 4% paraformaldehyde in phosphate buffered saline (PBS). The brains were immediately removed from the cranium and fixed for an additional 4 hr at 4°C. The brains were then cryoprotected by submersion in 18% (w/v) sucrose in PBS at 4°C overnight. The samples were embedded in OCT, frozen in powdered dry ice for 5 min, and then allowed to equilibrate to the cutting temperature (−20°C) of the cryostat (Microm Cryo-Star HM 560 Cryostat, Thermo Fisher Scientific, Inc., MA, USA). Frozen serial sections were sliced at 15 µm and allowed to air dry on gelatin-coated slides for hematoxylin and eosin staining and immunocytochemistry. For immunocytochemistry a primary antibody to tyrosine hydroxylase (Chemicon International Inc., CA, USA) was used at a dilution of 1∶500, and the secondary antibody (Alexa Fluor 568-conjugated goat anti-mouse IgG antibody; Invitrogen, ON, Canada) was used at a dilution of 1∶500.
